# Ontogenetic changes in root and shoot respiration, fresh mass and surface area of *Fagus crenata*

**DOI:** 10.1093/aob/mcac143

**Published:** 2022-12-26

**Authors:** Yoko Kurosawa, Shigeta Mori, Mofei Wang, Juan Pedro Ferrio, Tomohiro Nishizono, Keiko Yamaji, Kohei Koyama, Toshikatsu Haruma, Kohei Doyama

**Affiliations:** Faculty of Agriculture, Yamagata University, Tsuruoka, Yamagata, Japan; The United Graduate School of Agricultural Sciences, Iwate University, Morioka, Iwate, Japan; Faculty of Agriculture, Yamagata University, Tsuruoka, Yamagata, Japan; Faculty of Agriculture, Yamagata University, Tsuruoka, Yamagata, Japan; The United Graduate School of Agricultural Sciences, Iwate University, Morioka, Iwate, Japan; Aragon Agency for Research and Development (ARAID), Zaragoza, Spain; Department of Agricultural and Forest Systems and the Environment, Agrifood Research and Technology Centre of Aragon (CITA), Zaragoza, Spain; Department of Forest Management, Forestry and Forest Product Research Institute, Tsukuba, Ibaraki, Japan; Graduate School of Life and Environmental Sciences, University of Tsukuba, Tsukuba, Ibaraki, Japan; Department of Agro-environmental Science, Obihiro University of Agriculture and Veterinary Medicine, Obihiro, Hokkaido, Japan; Division of Sustainable Resources Engineering, Faculty of Engineering, Hokkaido University, Sapporo, Hokkaido, Japan; Graduate School of Life and Environmental Sciences, University of Tsukuba, Tsukuba, Ibaraki, Japan

**Keywords:** *Fagus crenata*, trade-off, metabolic scaling, allometry, ontogeny, root : shoot ratio, whole-plant mass, whole-plant respiration, whole-plant surface area, whole-root system

## Abstract

**Background and Aims:**

To date, studies on terrestrial plant ecology and evolution have focused primarily on the trade-off patterns in the allocation of metabolic production to roots and shoots in individual plants and the scaling of whole-plant respiration. However, few empirical studies have investigated the root : shoot ratio by considering scaling whole-plant respiration at various sizes throughout ontogeny.

**Methods:**

Here, using a whole-plant chamber system, we measured the respiration rates, fresh mass and surface area of entire roots and shoots from 377 *Fagus crenata* individuals, from germinating seeds to mature trees, collected from five different Japanese provenances. Non-linear regression analysis was performed for scaling of root and shoot respiration, fresh mass and surface area with body size.

**Key Results:**

Whole-plant respiration increased rapidly in germinating seeds. In the seedling to mature tree size range, the scaling of whole-plant respiration to whole-plant fresh mass was expressed as a linear trend on the log–log coordinates (exponent slightly greater than 0.75). In the same body size range, root and shoot respiration vs. whole-plant fresh mass were modelled by upward-convex (exponent decreased from 2.35 to 0.638) and downward-convex trends (exponent increased from –0.918 to 0.864), respectively. The root fraction in whole-plant respiration, fresh mass and surface area shifted continuously throughout ontogeny, increasing in smaller seedlings during early growth stages and decreasing in larger trees.

**Conclusions:**

Our results suggest a gradual shift in allocation priorities of metabolic energy from roots in seedlings to shoots in mature trees, providing insights into how roots contribute to shoot and whole-plant growth during ontogeny. The models of root : shoot ratio in relation to whole-plant physiology could be applied in tree growth modelling, and in linking the different levels of ecological phenomena, from individuals to ecosystems.

## INTRODUCTION

Allocation of metabolic production in plants is a complex process that reflects trade-offs among organs with different roles (Thornley, 1972; Shipley and Meziane, 2002; McCarthy and Enquist, 2007). Allocation to the structure and function of shoots may enhance carbon gain; however, it occurs at the expense of water uptake by roots, and vice versa. Optimal partitioning theory suggests that plants should allocate biomass to organs that require the most limiting resources in variable environments (Thornley, 1972; Bloom *et al*., 1985). Many studies have supported this theory; however, the allocation is also likely to be regulated by differences in plant size (Gedroc *et al*., 1996; Müller *et al*., 2000; Poorter *et al*., 2015; Ledo *et al*., 2018). The relationships between biomass allocation and plant size have been widely studied using allometric analyses. Several studies have suggested general allometric scaling rules for terrestrial plants that constrain allocation according to plant size (Enquist and Niklas, 2002; McCarthy and Enquist, 2007). Other studies suggest a continuous shift in the scaling exponent of such allometric relationships with plant size (Poorter *et al*., 2015). Nevertheless, few empirical studies have compared the metabolic rate or resource acquisition area between roots and shoots at the whole-plant level across a wide range of plant sizes, including mature trees. Thus, uncertainty regarding the allocation of metabolic production with body size remains, and how the combined effects of variation in body size and environmental conditions alter the root : shoot ratio is not fully understood.

Respiration provides energy for the growth of organisms; hence, elucidating the relationships between whole-plant respiration and plant size is crucial for terrestrial plant growth modelling (Sibly *et al*., 2012; O’Leary *et al*., 2019). For various organisms, metabolic rates have been conventionally assumed to scale with body mass to the ¾-power (Kleiber, 1932). However, the validity and universality of the ¾-power law of metabolic scaling remain controversial (West *et al*., 1997; Makarieva *et al*., 2008; Banavar *et al*., 2014; Glazier, 2018). Particularly for trees, measurement of whole-plant respiration is very rare and commonly estimated from partial respiration rates (Mori and Hagihara, 1988, 1991; Reich *et al*., 2006). To address this issue, Mori *et al*. (2010) developed an accurate method to measure the whole-plant respiration of trees including roots. They investigated the scaling of whole-plant respiration by measuring the whole-plant respiration of 183 excavated trees including roots across nine orders of magnitude in mass up to 10^4^ kg, with various tree species from Siberian to Southeast Asian biomes. They found that the scaling of whole-plant respiration indicated a shift in the scaling exponent (i.e. a slope on the log–log coordinates) from 1.41 to 0.81 in the transition from seedlings to mature trees. This finding has implications for physiochemical constraints imposed by gravity on the metabolic scaling of terrestrial plants (Atkin, 2010; Mori *et al*., 2010; Ballesteros and Luque, 2018; Kurosawa *et al*., 2021; Wang *et al*., 2021). The metabolic theory of ecology predicts that both plant productivity and biomass are size-dependent, and thus the physiochemical constraints will ultimately impact the dynamics of these factors (Sibly *et al*., 2012; Chave, 2013).

For Siebold’s beech, *Fagus crenata*, Kurosawa *et al*. (2021) reported a size-dependent increase of the root fraction in respiration (i.e. total root respiration divided by whole-plant respiration), fresh mass (i.e. total root fresh mass divided by whole-plant fresh mass) and surface area (i.e. total root surface area divided by whole-plant surface area) in the early growth stage from the current year (after expanding true leaves) to 1-year-old. Considering the high mortality rate of *F. crenata* in the early growth stage, preferential root growth may be an important process of enhancing water uptake and minimizing seedling death. Rapid root growth in the early growth stage is assumed to help enhance photosynthetic performance by increasing water and nutrient uptake. However, the relationship between plant size throughout ontogeny, from germinating seeds to mature trees, and respiration changes in the entire root system remains unclear.

The purpose of this study was to investigate the scaling of whole-plant respiration and surface area of *F. crenata* in over a broad size range, from germinating seeds to mature trees, by integrating empirical data of roots and shoots, and using individuals from various regions representative of different provenances. In the present study, we consistently used whole-plant closed chambers developed by Mori *et al*. (2010) and conducted each respiration measurement per individual tree to establish a reliable dataset that encompasses the entire course of ontogeny. *Fagus crenata* is widely distributed in Japan and often dominates typical cool-temperate deciduous forests (Fujii *et al*., 2002). The climatic conditions of the coastal areas in the Japan Sea and the Pacific Sea are characterized by heavy and light snowfall, respectively. Fujii *et al*. (2002) analysed chloroplast DNA haplotypes across the *F. crenata* distribution range and reported that the clades of this species are largely divided between the Japan Sea and Pacific Ocean sides of the Japanese Archipelago. Variations among the provenances or regions of *F. crenata* have also been reported to alter the morphological and physiological properties of the shoots, such as the transpiration rate, leaf area and leaf thickness (Bayramzadeh *et al*., 2008; Tateishi *et al*., 2010). In the present study, we used trees from various provenances and environments to show that the shoot–root balance changes dynamically during ontogeny depending on size. We suggest that the scaling of whole-plant respiration is generated from a size-dependent shift in the shoot–root balance. The findings from our empirical and novel approach can provide insights to better understand the mechanisms of tree growth based on whole-plant physiology and to evaluate the carbon budgets of forests.

## MATERIALS AND METHODS

### Plant materials and datasets

We used 377 *F. crenata* individuals in a wide range of sizes, with their developmental stage ranging from germinating seeds to mature trees of ~80 years of age. The plants originated from five provenances in Japan that have different climatic conditions (see Supplementary Data Fig. S1 for the geographical distribution of the five provenances and Table S1 for the number of materials by provenance). The whole-plant fresh mass of the individuals used for measurement of respiration was in the range 0.0000954–583 kg, while that for measurement of surface area was in the range 0.000413–10.2 kg (see Table S2).

To understand the scaling of whole-plant respiration with consideration of the balance between carbon acquisition and water uptake via true leaves and roots, we categorized plants into ‘germinating seeds–cotyledon stage’ and ‘seedlings (with true leaves)–mature stage’ groups. The ‘germinating seeds–cotyledon stage’ refers to the period when the seed has absorbed water and germinated – expanding the cotyledons but before the development of the first true leaves – during which individual growth is influenced partly by the seed reserves in the cotyledons. For the seedlings–mature stage, we separated individuals into shoot and root and measured their respiration rate, fresh mass and surface area. For the germinating seeds–cotyledon stage, we only measured whole-plant respiration and fresh mass of intact individuals.

The plants of the germinating seeds–cotyledon stage group were prepared by sowing the seeds collected in Yamagata. The sampling of seedlings–mature was conducted in a variety of growth environments, across large gradients of tree density and light availability, to account for the potential plasticity of respiration rates at various plant sizes. Therefore, both suppressed (in more light-limited conditions) and dominant (in sufficient light conditions) individuals were included in our measurements. The dataset included data reported by Mori *et al*. (2010) and Kurosawa *et al*. (2021), as well as the data obtained during this work (see Supplementary Data Table S2 for the data and their sources).

### Measurement of respiration rate, surface area and fresh mass

Measurement of respiration of individuals was conducted during the growing season, i.e. spring to summer for the seeds–cotyledon stage, and summer for the seedlings–mature stage. We sampled individual trees with carefully excavated entire root systems. Excavation of root systems in the field was performed in an area over twice the size of the canopy area and to a depth where the presence of roots was confirmed. For large trees, taproots were excavated using a grapple on heavy equipment, and the roots left in the soil were also excavated with the grapple and finally carefully collected using shovels. After excavation, any soil on the excavated roots was removed by washing. Individuals of the germinating seeds–cotyledon stage were left intact, while the seedlings–mature trees were cut into shoots and roots. For seeds to relatively small trees, the fresh mass of the whole plant, entire shoot and root was measured using a digital balance. For large trees, the fresh mass of entire shoots and roots was measured using a digital crane scale with a capacity of 2000 kg (Handy Cosmo II, Shuzui, Nagoya, Japan). After fresh mass measurement, trees were covered with a wet cloth to prevent transpiration and kept in a cool and dark place until measurement of their respiration rates (Reich *et al.*, 2006; Mori *et al*., 2010; Ferrio *et al*., 2018; Wang *et al*., 2021). The time from excavation to the end of respiration measurement per individual was about 5–30 min for germinating seeds to young trees, and several hours for large trees mainly due to the time-consuming excavation process.

**Fig. 1.**
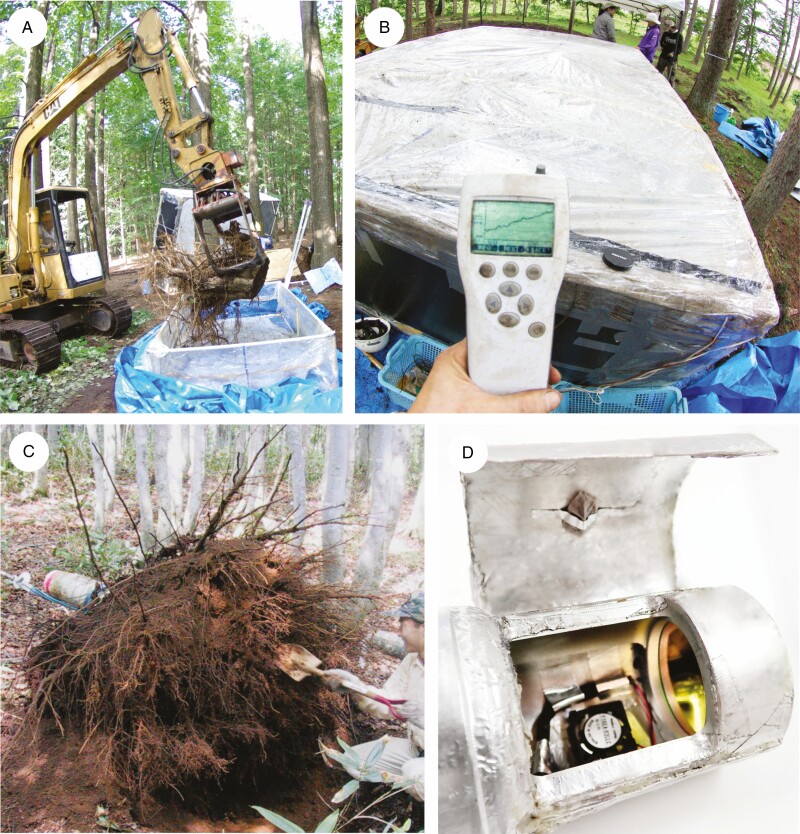
Measurement of whole-plant respiration using a closed-air circulation chamber. (A) Measurement of excavated whole roots from a mature tree. (B) Monitoring of CO_2_ increment by respiration of mature tree material in the chamber. (C) Roots of a mature tree immediately after excavation. (D) Measurement of the germinating seeds, using the smallest chamber (80 cm^3^). The seeds are wrapped in a wet sheet and attached to the top cover of the chamber. A small fan is attached to the inside of the sensor head.

Respiration measurement was conducted on the entire shoot and root of the individuals for the seedlings–mature stage, and on the intact individuals for the seeds–cotyledon stage. For respiration measurement, we used the methods developed by Mori *et al*. (2010) (Fig. 1). Respiration at the whole-plant level does not change before or after tree excavation (Mori *et al*., 2010). We enclosed the plant materials in customized incubation chambers and measured the CO_2_ concentrations within the chambers every 5 s for approximately 30–300 s with a CO_2_ probe (GMP343, Vaisala, Helsinki, Finland). During the measurements, the CO_2_ concentrations in the chamber were homogenized using forced air circulation with DC axial fans and increased linearly with measurement time. To obtain accurate measurements within a short period, a chamber that is too large for the materials should not be used. Therefore, suitable chamber sizes were selected from ten chambers of various volumes (80 cm^3^ to 8 m^3^) according to the plant sizes. All the chambers were confirmed to be sufficiently airtight by leak testing. In large-volume chambers, a cylindrical air duct attached to the fans facilitated sufficient air circulation. During the measurements, temperature variation in the chambers within 1 °C was observed. To reconcile temperature differences among respiration measurements, we adjusted all of the respiration rates to 20 °C, assuming *Q*_10 _= 2 (Atkin *et al*., 2005).

The measurement of whole-plant surface area was performed on seedlings to young trees (0.000413–10.2 kg in whole-plant fresh mass). Shoot surface area was calculated as the sum of the one-sided leaf area and surface areas of the stems and branches. Leaf area was measured using a leaf area meter (LI-3100C; LICOR, Lincoln, NE, USA). The surface areas of the stems and branches were calculated from their diameter and length, assuming that they were cylindrical or truncated cones (Whittaker and Woolwell, 1967; Mori and Hagihara, 1988). Root surface area was evaluated using the image analysis software WinRhizo version 2016a (Regent Instruments, Quebec, Canada). The root images for analysis were obtained using a flatbed scanner (Epson Perfection V800, Seiko Epson, Japan) at a resolution of 800 d.p.i. When the root diameter exceeded 2 cm, the root surface area was calculated from their diameter and length, assuming that they were cylindrical or truncated cones.

### Data analysis

The relationships between respiration rate (μmol CO_2_ s^–1^), fresh mass (kg) and surface area (m^2^) were plotted on the log–log coordinates, and two types of trends were observed: linear and curve (convex upward or downward) trends, with the latter implying a gradual change of the scaling exponent. Using these trends as candidates, we selected a model for each scaling relationship. The linear model on log–log coordinates is expressed as a simple power function as:


$$\begin{array}{l} \begin{array}{l} Y\;=\;FM^{\;f} \end{array} \end{array}$$ (1)

where *M* is the explanatory variable (e.g. fresh mass or surface area), *Y* is the response variable (e.g. respiration rate), *F* is the intercept and *f* is the scaling exponent (slope on the log–log coordinates). The convex trends on the log–log coordinates are expressed as follows (Shinozaki, 1979):


$$\begin{array}{l} \begin{array}{l} \frac{1}{Y}\;=\;\frac{1}{GM^{\;g}}\;+\;\frac{1}{HM^{\;h}}\;(g\;>\;h), \end{array} \end{array}$$ (2)

or


$$\begin{array}{l} \begin{array}{l} Y\;=\;GM{\;}^{g}\;+\;HM^{\;h}\;(g\;>\;h), \end{array} \end{array}$$ (3)

where *G* and *H* are the coefficients, while *g* and *h* are the exponents. As *M* varies, they approach the following asymptotic relationships:


$$\begin{array}{l} \begin{array}{l} Y\;=\;GM^{\;g} \end{array} \end{array}$$ (4)

$$\begin{array}{l} \begin{array}{l} Y\;=\;HM^{\;h}. \end{array} \end{array}$$ (5)

When $GM{\;}^{g}\;\ll \;HM{\;}^{h}$, eqn (2) approaches eqn (4), and eqn (3) approaches eqn (5). When $GM{\;}^{g}\;\gg \;HM^{\;h}$, eqn (2) approaches eqn (5), and eqn (3) approaches eqn (4). We used these functions because when *h* = 0 and log*M *= time, they translate into logistic functions, which are often the case in biological phenomena (Shinozaki, 1979). When point P on the log–log coordinates denotes the intersection between the two asymptotic lines, eqns (4) and (5), the value of *M* at point P is calculated as ${\left(H/G\right)}^{\left[1/(g-h)\right]}$. At $M\;=\;{\left(H/G\right)}^{\left[1/(g-h)\right]}$, the slope of the line that is tangent to eqns (2) and (3) is (g+h)/2 (Mori *et al*., 2010).

The regression analysis was performed using the Levenberg–Marquardt algorithm (LMA) (Elzhov *et al*., 2013) using the nlsLM function in the minpack.lm package in R (R Core Team, 2019). We compared the three models using Akaike’s information criterion (AIC) and deemed the model with the lowest AIC as the best fit (Burnham and Anderson, 2004). Using the Bayesian information criterion (BIC) did not affect our results. In the case where the linear model was the best, we conducted a reduced major axis (RMA) regression analysis (Niklas and Hammond, 2014) of the log-transformed version of eqn (1) using the PAST analysis software (Hammer *et al*., 2001). RMA regression analysis was also performed to test the differences among the provenances in the exponents for scaling of whole-plant respiration as well as root and shoot respiration to whole-plant fresh mass. RMA regression minimizes collective distance between data points and the fitted line, assuming that the error variance of the *X* and *Y* variables is the same, relative to the total variance on each axis. This approach is considered appropriate when the variables on both axes may be measured with error, and when the purpose of a study is to describe how two variables are related (Smith, 2009). Finally, using the obtained functions for the relationships of root and shoot to whole-plant fresh mass, we tested how the root fraction (roots/whole-plant, %) shifted in the seedlings–mature stage, with respect to respiration, fresh mass and surface area.

## RESULTS

### Whole-plant respiration in germinating seeds to mature trees

In our dataset, the whole-plant fresh mass for the germinating seeds–cotyledon stage was in the range 0.0000954–0.000470 kg (*n* = 39), while that for the seedlings (with true leaves)–mature stage was 0.000276–583 kg (*n* = 267; see Supplementary Data Table S2).

**Fig. 2.**
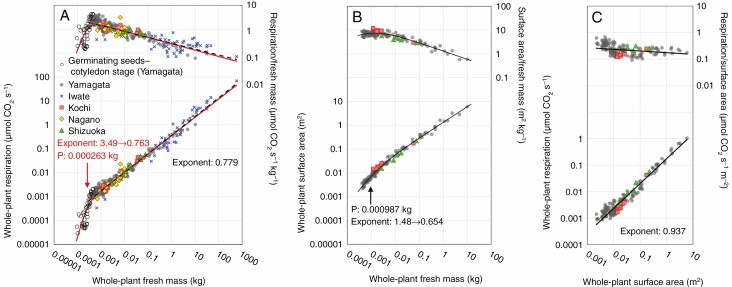
Scaling of whole-plant respiration vs. fresh mass (A), whole-plant surface area vs. fresh mass (B) and whole-plant respiration vs. surface area (C). The respiration rates were adjusted to 20 °C. The solid lines in the figures indicate the best-fit model for each relationship (red: germinating seeds–cotyledon stage, black line: seedlings–mature stage). The dashed line in A shows the RMA fit for the seedlings to mature stage (for this relationship, model selection was not conducted). For models of curve trends, changes in the scaling exponent with increasing plant size are shown. Point P represents the fresh mass at the asymptotes’ intersection of the curve trends. The dataset of germinating seeds–cotyledon stage in A is not included in B and C, or in Figs 3–5.

The datasets for the two growth stages were combined for the scaling of whole-plant respiration in the ontogeny from seed germination to mature trees. In the size range from germinating seeds to mature trees, the relationship between whole-plant respiration and whole-plant fresh mass showed a convex upward trend on the log–log coordinates in eqn (2) (*n* = 306, red line in Fig. 2A, see Supplementary Data Table S3 for AIC). With increasing plant mass, the exponent decreased from 3.49 to 0.763. The whole-plant fresh mass at the asymptotes’ intersection point P was 0.000263 kg. The observations indicate that the scaling exponent was quite high, exceeding 3.0 in the early growth stage from seed germination to before true leaves developed, and decreased gradually to nearly 0.75 in mature trees. Furthermore, the observed whole-plant respiration of *F. crenata* for germinating seeds to mature trees was within the range reported for whole-plant respiration in seedlings to mature trees of 52 species from biomes in Siberia to Southeast Asia (Mori *et al*., 2010), which was modelled according to the same upward convex trend (see Fig. S2).

Note that the results described below do not include the data of the seeds–cotyledon stage. For the dataset of the seedlings–mature stage, the scaling of whole-plant respiration was analysed by a linear fit (*n* = 267, black dashed line in Fig. 2A). In ordinary least square (OLS) regression, the scaling exponent was 0.769 [95 % confidence interval (CI): 0.751–0.789] and the intercept was 0.355 (95 % CI: 0.320–0.399). In RMA regression, the exponent was 0.779 (95 % CI: 0.762–0.797) and the intercept was 0.371 (95 % CI: 0.337–0.413). These were obtained with a satisfactory coefficient of determination value (*R*^2^ = 0.975). Therefore, considering the ontogeny from seedlings to mature trees, scaling of whole-plant respiration to whole-plant fresh mass in our results was well expressed by a linear trend, with the scaling exponent slightly greater than 0.75. We also conducted RMA regression analysis within the range 0.001–0.1 kg, where the data for the five provenances were available, and observed that the difference in the scaling exponent among the provenances was not significant (Supplementary Data Fig. S3 and Table S4). The results suggest that the differences in the size response of whole-plant respiration among the provenances were not significant in the size ranges of the seedlings.

For the relationships between whole-plant surface area and whole-plant fresh mass, eqn (2) had the best fit with a convex upward trend (Fig. 2B, the whole-plant surface area ranged between 0.00225 and 5.89 m^2^, and the whole-plant fresh mass ranged between 0.000413 and 10.2 kg, *n* = 154). In the trend, the exponent shifted from 1.48 to 0.654 with increasing whole-plant fresh mass, and the intersection point P was 0.000987 kg. For the relationships between whole-plant respiration and whole-plant surface area, the best model was the linear trend of eqn (1) (Fig. 2C; see Supplementary Data Table S3 for AIC, *n* = 152). RMA regression analysis had an intercept of 0.174 (95 % CI: 0.157–0.200) and a scaling exponent of 0.937 (95 % CI: 0.908–0.971) for *R*^2^ = 0.940, suggesting consistently negative allometry (exponent <1.0).

### Respiration of shoots and roots in seedlings to mature trees

We analysed the respiratory scaling in shoot and root with their fresh mass and surface area in the size range of seedlings to mature trees (between 0.000413 and 10.2 kg in whole-plant fresh mass).

**Fig. 3.**
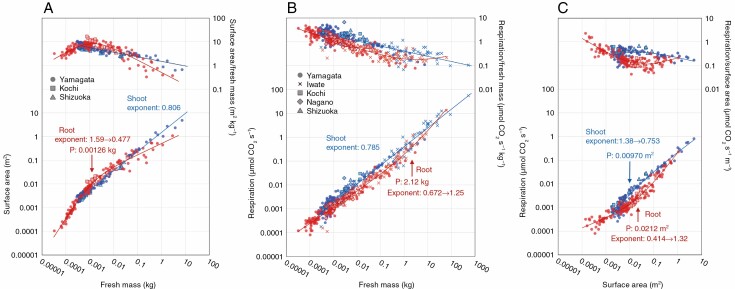
Scaling of surface area vs. fresh mass (A), respiration vs. fresh mass (B) and respiration vs. surface area (C) in shoot (blue) and root (red). For models of curve trends, changes in the scaling exponent with increasing fresh mass and surface area are shown. Point P represents the fresh mass and surface area at the asymptotes’ intersection of the curve trends.

The relationships between surface area vs. fresh mass in shoot and root are shown in Fig. 3A with fitting lines of the trend with the lowest AIC (see Supplementary Data Table S5 for AIC). The shoot surface area vs. shoot fresh mass was fitted best by the linear trend of eqn (1) (shoot surface area ranged between 0.0017 and 4.81 m^2^, and shoot fresh mass ranged between 0.000287 and 7.29 kg, *n* = 157). For this relationship, RMA regression analysis provided a scaling exponent of 0.806 (95 % CI: 0.783–0.829) and intercept of 1.49 (95 % CI: 1.27–1.74) for *R*^2^ = 0.985. In contrast, root surface area vs. root fresh mass was fitted best by eqn (2), describing a convex upward trend (root surface area ranged from 0.000103 to 1.08 m^2^ and root fresh mass ranged between 0.0000330 and 2.91 kg, *n* = 164). With increasing root fresh mass, the scaling exponents shifted from 1.59 to 0.477. The root fresh mass at the intersection point P was 0.00126 kg, which was estimated to correspond to 0.00206 kg of whole-plant fresh mass according to the best model described below. This indicates that the mass-specific surface area in the shoots declines continuously during ontogeny, while that in roots increases and then decreases, according to plant size.

Shoot respiration vs. shoot fresh mass was fitted best by the linear trend of eqn (1) (Fig. 3B, shoot fresh mass ranged between 0.000210 and 521 kg, *n* = 268) (see Supplementary Data Table S5 for AIC). For this relationship, RMA provided an exponent of 0.785 (95 % CI: 0.768–0.805) and intercept of 0.403 (95 % CI: 0.355–0.459) in *R*^2^ = 0.968. In contrast, shoot respiration vs. shoot surface area was best fitted by eqn (2), describing a convex-downward trend (Fig. 3C, shoot surface area ranged between 0.0017 and 4.81 m^2^, *n* = 155). In the relationship, the scaling exponents shifted from 1.38 to 0.753 with increasing shoot surface area, and the shoot surface area at the intersection point P was 0.00970 m^2^. This was estimated to correspond to 0.00671 kg at the whole-plant fresh mass.

Root respiration vs. root fresh mass was fitted best by eqn (3), describing a convex-downward trend (Fig. 3B, root fresh mass ranged from 0.000033 to 62.1 kg, *n* = 267) (see Supplementary Data Table S5 for AIC). The exponent shifted from 0.672 to 1.25 with increasing root fresh mass. The root fresh mass at intersection point P was 2.12 kg, which was estimated to be 11.74 kg of the whole-plant fresh mass. In addition, root respiration vs. root surface area was also fitted best by eqn (3), describing a convex-downward trend (Fig. 3C, root surface area ranged from 0.000103 to 1.08 m^2^, *n* = 162). The scaling exponent shifted from 0.414 to 1.32. The root surface area at intersection point P was 0.0212 m^2^, which was estimated to correspond to 0.00369 kg of the whole-plant fresh mass.

### Ontogenetic shift in root fraction of the whole-plant in seedlings to mature trees

**Fig. 4.**
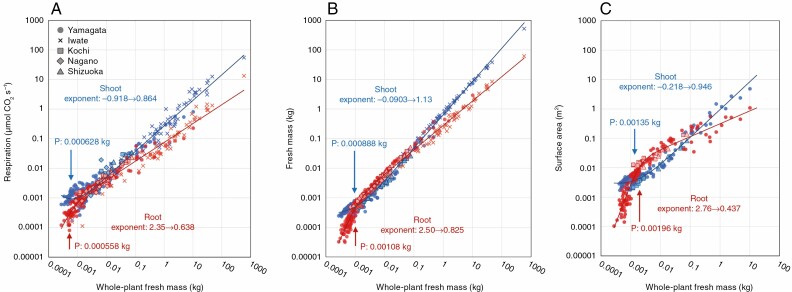
Relationships between shoot (blue) and root (red) respiration rate (A), fresh mass (B) and surface area (C) in relation to whole-plant fresh mass. For each relationship, the change in the scaling exponent with increasing whole-plant fresh mass is shown. Point P represents the fresh mass at the asymptotes’ intersection of the curve trends.

To analyse the ontogenetic shift in root–shoot balance from seedlings to mature trees, we conducted model selection for scaling of the shoot and root respiration, fresh mass, and surface area to whole-plant fresh mass. The log–log plots of shoot and root to whole-plant fresh mass are shown in Fig. 4 with fitting lines of the trend with the lowest AIC (see Supplementary Data Table S6 for AIC). We also conducted RMA regression analysis within 0.001–0.1 kg, and differences in the scaling exponent among provenances were not significant (Fig. S4 and Table S7).

The relationships between shoot respiration rate, fresh mass, and surface area and whole-plant fresh mass showed a convex-downward trend in eqn (3). In all cases, the scaling exponents for shoots shifted from <0 in small plants to >0.8 in large plants. The exponent shifted from −0.918 to 0.864 for respiration (*n* = 267), from −0.0903 to 1.13 for fresh mass (*n* = 337), and from −0.218 to 0.946 for surface area (*n* = 157). The values of whole-plant fresh mass at point P were 0.000628, 0.000888 and 0.00135 kg for the shoot respiration rate, shoot fresh mass and shoot surface area, respectively.

The relationships between root respiration rate, fresh mass, and surface area and whole-plant fresh mass were convex-upward in eqn (2). In all cases, the scaling exponent shifted from >2 in small plants to <1 in large plants. The exponent shifted from 2.35 to 0.638 for respiration (*n* = 267), from 2.50 to 0.825 for fresh mass (*n* = 337), and from 2.76 to 0.437 for surface area (*n* = 164). The values of whole-plant fresh mass at point P were 0.000558, 0.00108 and 0.00196 kg for the root respiration rate, root fresh mass and root surface area, respectively.

**Fig. 5.**
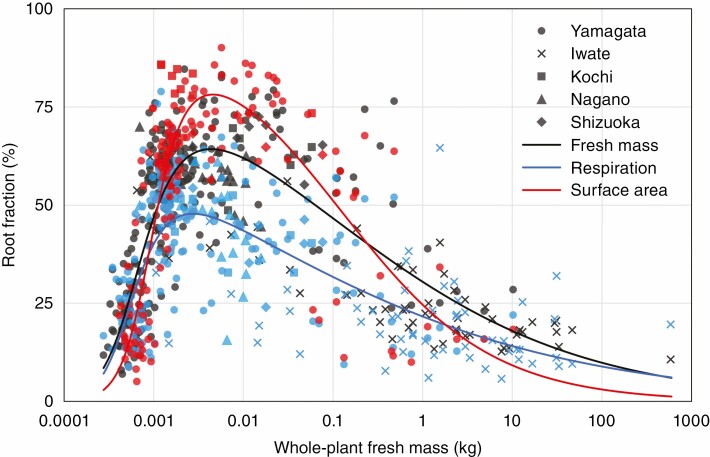
Plot diagram of the root fraction (%) of respiration rates, fresh mass and surface area of *F. crenata* in relation to whole-plant fresh mass from seedlings to mature stages. Blue: respiration rates (*n* = 267); black: fresh mass (*n* = 337); red: surface area (*n* = 154).

Using the obtained functions for the relationships between shoot and root to whole-plant fresh mass shown in Fig. 4, we calculated the root fraction (root/whole-plant, %) from seedlings to mature trees (Fig. 5). Overall, the root fraction significantly increased in small seedlings and gradually decreased in larger plants with increasing plant size. Calculated at 10-mg intervals, the peak of root fraction was 47.8 % at 0.00274 kg for respiration rate, 64.3 % at 0.00436 kg for fresh mass, and 78.2 % at 0.00458 kg for surface area. These results indicate that the allocation of metabolic products is preferred for roots during the early growth stage, and gradually shifts to shoots in mature trees.

## DISCUSSION

For the size range from germinating seeds to mature trees, a convex-upward trend was observed for the whole-plant respiration to whole-plant fresh mass (red line in Fig. 2A). The scaling exponent decreased gradually from >3.0 during seed germination to ~0.75 in mature trees. In the initial growth stage, from seed germination to before expansion of true leaves, photosynthesis is not fully developed when compared with after the true leaves developed. For the initial growth stages, Huang *et al*. (2020*a*) reported a steep increase in mass-specific respiration after a water-triggered break in seed dormancy and strong constraints by water content on the respiration rate. Therefore, the observed high scaling exponents for the early growth stage are probably caused by the water content constraint, rather than plant size. For *F. crenata*, it seems that once plants become independent of seed storage and develop structures and functions for carbon gain and water uptake, the exponent decreases and approaches ~0.75. Furthermore, the whole-plant respiration in *F. crenata* from germinating seeds to mature trees was within the range of whole-plant respiration from seedlings to mature trees in 51 species by Mori *et al*. (2010) (Supplementary Data Figure S2). In the inter-specific scaling by Mori *et al*. (2010), the exponent was shown to decrease from 1.41 in seedlings to 0.81 in large trees. The results suggest that after germination of seeds in various size ranges and development of the shoot and root systems, whole-plant respiration of various tree species converges according to body mass, with scaling exponents of ~0.75–0.8 in large trees.

Notably, respiration rates, fresh mass and surface areas of whole plants, shoots and roots seem to considerably converge along their sizes, even though the dataset included trees from various provenances with different climatic conditions (Figs 2–4). For the size range of seedlings, statistical analysis results showed that the differences among provenances in the scaling of whole-plant respiration were not significant (Supplementary Data Fig. S3 and Table S4). Similarly, the differences among provenances in the scaling of shoots and roots (in respiration, fresh mass and surface area) with body size in the size range of seedlings were not significant (Fig. S4 and Table S7). The findings suggest that, despite the intraspecific variation in single-leaf level morphology and physiology (Bayramzadeh *et al*., 2008; Tateishi *et al*., 2010), as well as gene phylogeny (Fujii *et al*., 2002), variation in the structure and function of entire shoots and roots are limited to a certain range based on plant size during early growth stages. This could be partly because of physicochemical constraints, such as gravity, on shoot and root respiration. In the present study, owing to data limitation, the effects by provenance on respiration rates were not clarified over a wide plant size range. However, considering scaling of whole-plant respiration was consistent between *F. crenata* and other various species over a wide range of plant size (Fig. S2), it is possible that shoot and root respiration are consistent among tree species. Consequently, the trade-off relationship in energy use between shoot and root may contribute to the establishment of the baseline of the scaling of whole-plant respiration in terrestrial plants. This should be tested with further investigations on intra- and inter-specific scaling of shoot and root respiration.

For the seedling to mature tree size range, consistent with previous empirical studies (Cheng *et al*., 2010; Mori *et al*., 2010; Huang *et al*., 2020*b*), the scaling of shoot respiration to shoot fresh mass exhibited negative allometry (Fig. 3B), potentially because of the accumulation of inactive tissue through ontogeny. In contrast, the convex-downward trend in root respiration with root fresh mass and surface area (Fig. 3B, C) was unexpected. The convex-downward trend in root respiration is clearer in relation to surface area than to fresh mass. This was because of the ontogenetic shift in the mass-specific root surface area, which increased in the early growth stage and decreased in mature trees (Fig. 3A). The decrease in mass-specific root surface area indicates increased taproot diameter from root collar, which would provide structural support for the shoots in large trees. However, robustness of the scaling of root respiration should be further investigated, because we used relatively small sample sizes of root for mature trees. In addition, despite our careful excavation procedure, fine roots might be lost during excavation, especially from the root systems of mature trees. Further measurement of root systems of mature trees and precise estimation of the amount of the lost roots would provide a better understanding of the respiration–mass–surface area relationships for root systems.

In the seedling to mature tree plant size range, whole-plant respiration with whole-plant fresh mass was well expressed as a linear trend (black dashed line in Fig. 2A). In contrast, shoot and root (in respiration, fresh mass and surface area) showed contrasting and clear convex trends based on plant size on the log–log coordinates, with downward- and upward-convex curves, respectively (Fig. 4). The whole-plant fresh mass at the asymptotes’ intersection point P for root and shoot was observed in the early growth stage (<0.002 kg, Fig. 4), suggesting that the metabolic product in plants is preferentially invested in roots during the early growth stage immediately after germination, and subsequently for shoots at later stages up to mature trees (Fig. 5). During the early growth stage, the root fraction increased significantly up to 78.2 % in surface area, and only to 47.8 % in respiration. This is consistent with the finding of Kurosawa *et al*. (2021). As they suggested, it is probable that the rapid and energetically efficient root growth may support survival of seedlings in the early growth stage, which are typically characterized by high mortality rate, and promote establishment of *F. crenata* forests.

For seedlings in the early growth stage (<0.002 kg in whole-plant fresh mass), we also observed that shoot respiration, fresh mass and surface area decreased with increasing plant size, as evidenced by exponents <0 (Fig. 4). This may be because of a decrease in cotyledons (seed reserves), which are the main energy source for rapid root development in small seedlings whose photosynthetic capacity has not yet been fully developed (Ampofo *et al*., 1976; Kitajima, 2002). Conversely, the subsequent preferential increase in shoots suggests an increase in photosynthetic capacity. This shift is consistent with the general perception of the tree growth model in that the increase in body size, i.e. dry mass, is relatively small in the early growth phase and growth is enhanced later (Weiner and Thomas, 2001). Therefore, our results indicate that the initial slow growth stage is characterized by preferential root growth to accelerate photosynthetic performance and subsequent shoot growth.

It has been widely recognized that overall plant growth is expressed as sigmoid curves, wherein the initial growth rate is low, subsequently exponentially increases, and finally declines toward asymptote (Tjørve and Tjørve, 2010). However, the physiological mechanisms that cause the decline in growth with increasing size after the mature growth stage have not been fully elucidated and remain controversial (Weiner and Thomas, 2001). We measured trees up to the mature and large size at which individual growth of *F. crenata* would begin to decline (Ono *et al*., 2013; Bourque *et al*., 2019) and asymptote would be observed for roots followed by the shoot (Fig. 4). The asymptote in root growth during the mature stage may cause an imbalance between water uptake and carbon acquisition, and consequently reduce shoot and whole-plant growth. This highlights the possibility that the ontogenetic shift in the shoot–root balance is one of the backgrounds of the *F. crenata* whole-plant growth model. However, it remains unclear how provenances, environments and their interaction influence the ontogenetic shifts in shoot–root balance and whole-plant respiration during ontogeny, which should be elucidated in future using experimental studies in common gardens and over wider plant size ranges. With an enhanced understanding of the effects of environments and genotypes (provenances), the models of ontogenetic scaling in shoot–root balance could be applied to general whole-plant growth models.

In summary, the contribution of the root system to the successful growth of individuals may include physiological support during early growth and may shift to physical support in mature trees. Furthermore, the ontogenetic shift in the root/shoot ratio indicates shifts in the balance between water uptake and carbon acquisition in individuals, in addition to shifts in whole-plant structures and functions. The capacity for water uptake and carbon acquisition at the individual level from seedlings to mature trees needs elucidation. The findings would facilitate the elucidation of the underlying factors constraining chronological changes in the carbon budget of forests, including the roots.

## SUPPLEMENTARY DATA

Supplementary data are available online at https://academic.oup.com/aob and consist of the following. Figure S1: geographical distribution of the five provenances where the individual *Fagus crenata* used for the current study measurements were found. Figure S2: comparison of the scaling of whole-plant respiration between *F. crenata* and 51 other tree species. Figure S3: comparison of the scaling of whole-plant respiration among provenances. Figure S4: comparison of the scaling relationships between shoot and root respiration, fresh mass, and surface area vs. whole-plant fresh mass among provenances. Table S1: sample size per provenance for the measurement of respiration, surface area and fresh mass. Table S2: datasets of respiration, surface area and fresh mass. Table S3: results of fitting analysis for the scaling of whole-plant respiration rates and whole-plant surface area. Table S4: results of reduced major axis regression for scaling of whole-plant respiration vs. fresh mass for each provenance within the mass range 0.001–0.1 kg. Table S5: results of fitting analysis for the scaling relationships among respiration, surface area and fresh mass for shoots and roots. Table S6: results of fitting analysis for the scaling of shoot and root respiration, fresh mass, and surface area vs. whole-plant fresh mass. Table S7: results of RMA regression for scaling of shoot and root respiration, fresh mass, and surface area vs. whole-plant fresh mass for each provenance.

mcac143_suppl_Supplementary_Figures

mcac143_suppl_Supplementary_Table_S1

mcac143_suppl_Supplementary_Table_S2

mcac143_suppl_Supplementary_Table_S3

mcac143_suppl_Supplementary_Table_S4

mcac143_suppl_Supplementary_Table_S5

mcac143_suppl_Supplementary_Table_S6

mcac143_suppl_Supplementary_Table_S7
