# Supplementary material for: Ontogenetic changes in root and shoot respiration, fresh mass and surface area of Fagus crenata
Source: Ann Bot. 2022 Dec 26;131(2):313–22. doi: 10.1093/aob/mcac143 (PMC9992930; doi:10.1093/aob/mcac143)
Supplement: mcac143_suppl_Supplementary_Figures [file mcac143_suppl_supplementary_figures.docx]

## Supplementary Figures


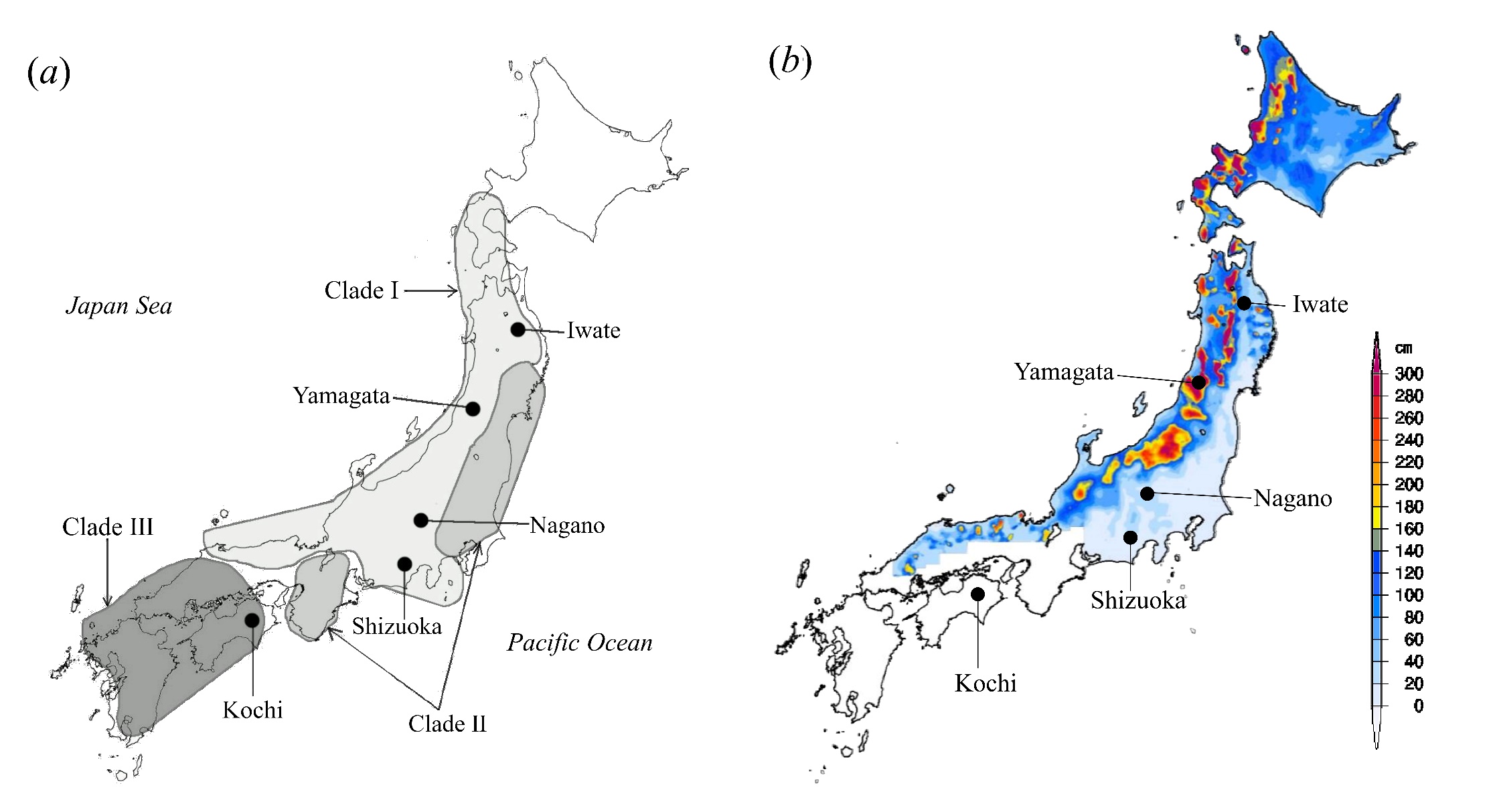


Figure S1. Five provenances where the individual *Fagus crenata* used for the current study measurements are located. (*a*) Three *F. crenata* clades based on the chloroplast DNA analysis. (*b*) Geographical variation in annual maximum snow depths in Japan. Two major clades (I and II+III) have been revealed in phylogenetic analyses among the haplotypes of *F. crenata*. Figure (*a*) redrawn from Fujii et al. (2002). Figure (*b*) is based on “Maximum depth of snow cover/Annual” (from the Japan Meteorological Agency website).

**Reference**

Fujii, N., et al. (2002). Chloroplast DNA phylogeography of *Fagus crenata* (Fagaceae) in Japan. *Plant Systematics and Evolution,* 232, 21–33. https://doi.org/10.1007/s006060200024


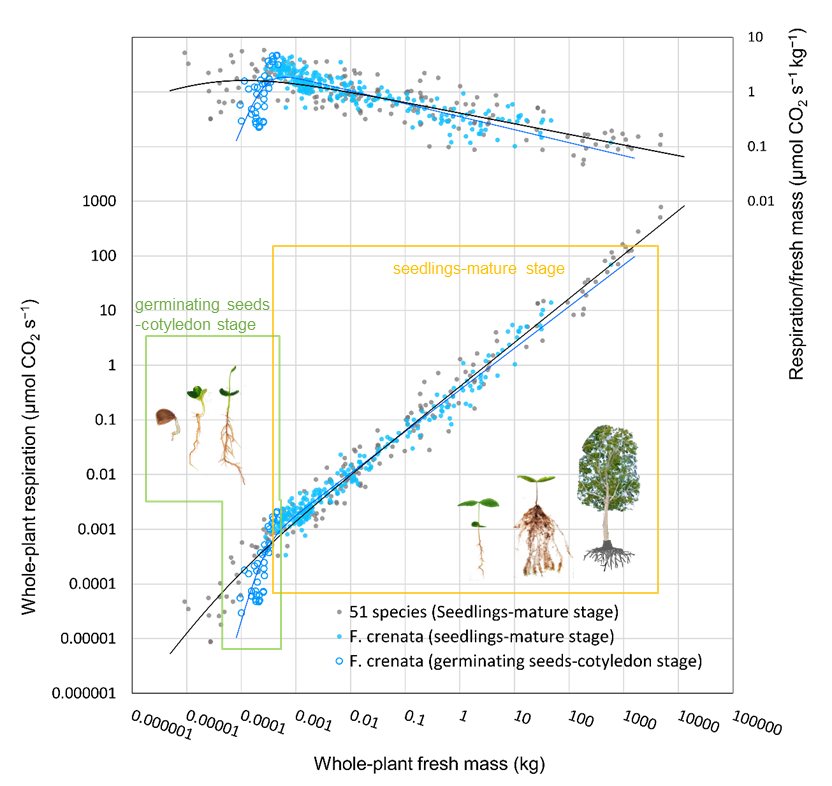


Figure S2. Comparison of scaling of whole-plant respiration in *Fagus crenata* and 51 other tree species. Black circles: data set from Mori et al. (2010) for 51 tree species from seedlings to mature trees, except *F. crenata*. Blue open circles: *F. crenata*, from germinating seeds to the seedlings without developed true leaves in the present study*.* Blue filled circles: *F. crenata*, from seedlings with developed true leaves to mature trees in the present study. Black line: best model in Mori et al. (2010). Blue line: best model for *F. crenata* the of germinating seeds–mature stage in the present study.

**Reference**

Mori, S., *et al.* (2010). Mixed-power scaling of whole-plant respiration from seedlings to giant trees. *Proceedings of the National Academy of Sciences of the United States of America,* 107, 1447–1451. https://doi.org/10.1073/pnas.0902554107


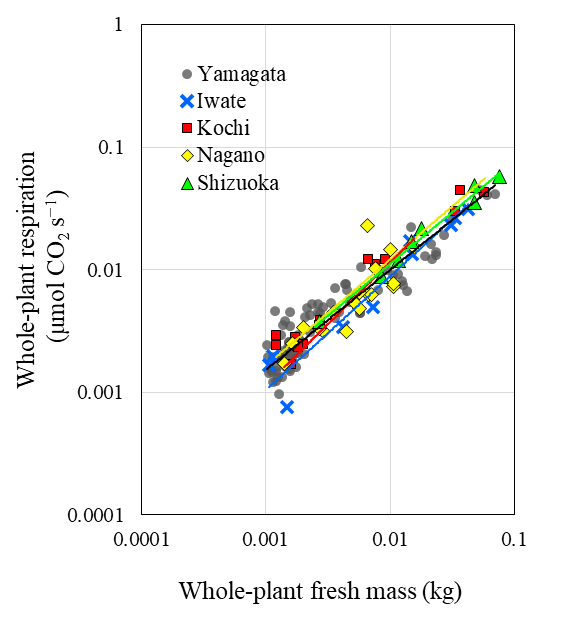


Figure S3. Comparison of the scaling relationships whole-plant respiration and whole-plant fresh mass among provenances by reduced major axis (RMA) regression analysis. Details are compiled in Table S4.


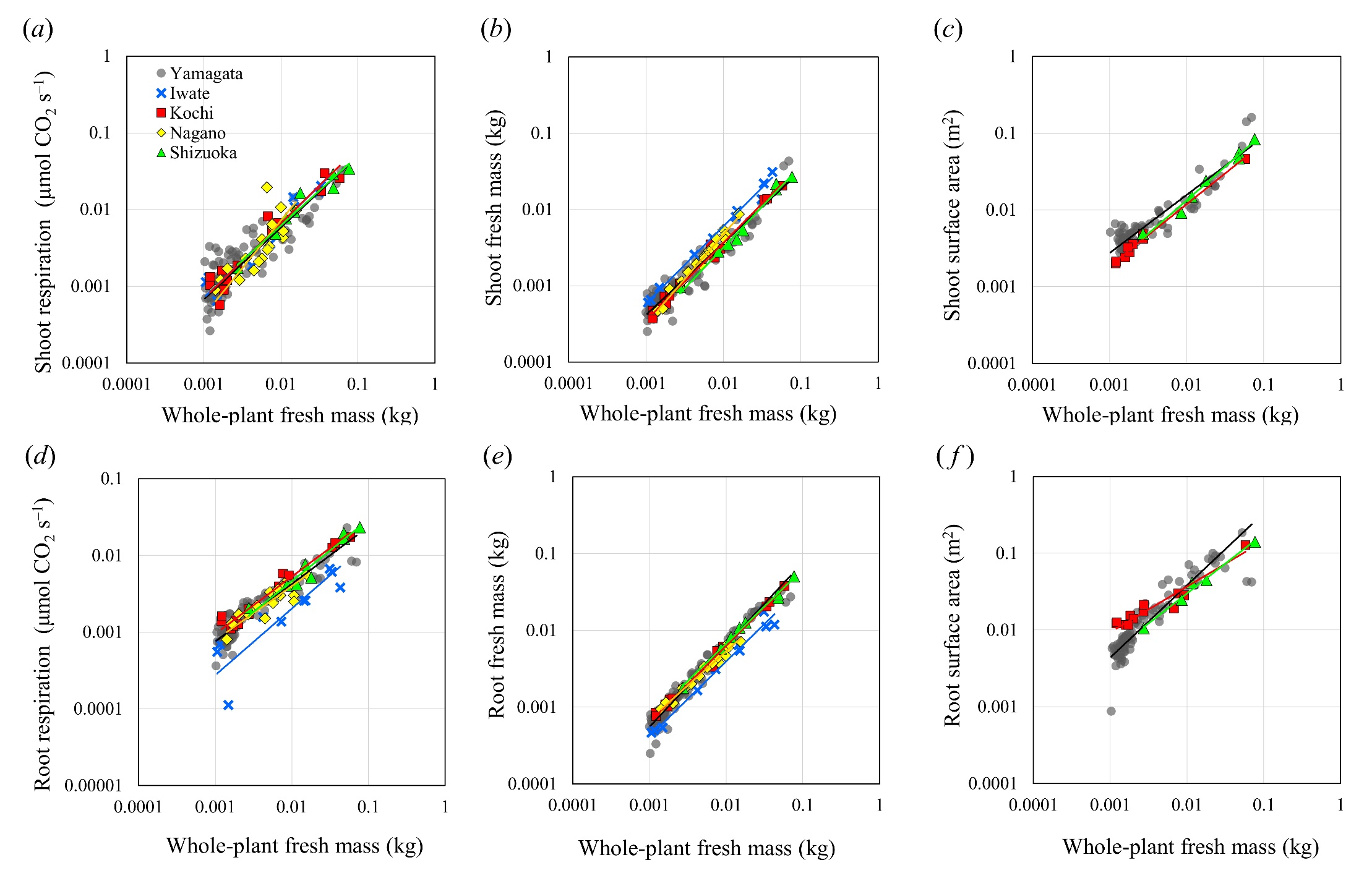


Figure S4. Comparison of the scaling relationships between respiration, fresh mass, and surface area of the shoot (*a*–*c*) and root (*d*–*f*) in relation to whole-plant fresh mass among provenances by reduced major axis (RMA) regression analysis. Details are compiled in Table S7.
