# Supplementary material for: Ontogenetic changes in root and shoot respiration, fresh mass and surface area of Fagus crenata
Source: Ann Bot. 2022 Dec 26;131(2):313–22. doi: 10.1093/aob/mcac143 (PMC9992930; doi:10.1093/aob/mcac143)
Supplement: mcac143_suppl_Supplementary_Table_S1 [file mcac143_suppl_supplementary_table_s1.docx]

Table S1. Sample size (*n*) per provenance for the measurement of respiration, surface area, and fresh mass.

| Growth stage | Provenance | Whole-plant fresh mass | Shoot fresh mass | Root fresh mass | Whole-plant respiration | Shoot respiration | Root respiration | Whole-plant surface area | Shoot surface area | Root surface area |
| --- | --- | --- | --- | --- | --- | --- | --- | --- | --- | --- |
| Germinating seeds–cotyledon stage | Yamagata | 39 | 0 | 0 | 39 | 0 | 0 | 0 | 0 | 0 |
| Seedlings–mature stage | Yamagata | 236 | 236 | 236 | 166 | 166 | 166 | 140 | 141 | 147 |
|  | Iwate | 58 | 59 | 58 | 58 | 59 | 58 | 0 | 0 | 0 |
|  | Kochi | 14 | 14 | 14 | 14 | 14 | 14 | 9 | 9 | 12 |
|  | Shizuoka | 8 | 8 | 8 | 8 | 8 | 8 | 5 | 7 | 5 |
|  | Nagano | 21 | 21 | 21 | 21 | 21 | 21 | 0 | 0 | 0 |
| Total |  | 376 | 338 | 337 | 306 | 268 | 267 | 154 | 157 | 164 |
