# Supplementary material for: Ontogenetic changes in root and shoot respiration, fresh mass and surface area of Fagus crenata
Source: Ann Bot. 2022 Dec 26;131(2):313–22. doi: 10.1093/aob/mcac143 (PMC9992930; doi:10.1093/aob/mcac143)
Supplement: mcac143_suppl_Supplementary_Table_S3 [file mcac143_suppl_supplementary_table_s3.docx]

Table S3. Results of fitting analysis and goodness of fit (AIC and BIC statistics) for the scaling of whole-plant respiration rates with whole-plant fresh mass and whole-plant surface area (Figure 2A–C).

| Growth stage in dataset | Relationship | Equation | Trend | AIC | BIC | *F* | *G* | *H* | *f* | *g* | *h* |
| --- | --- | --- | --- | --- | --- | --- | --- | --- | --- | --- | --- |
| Germinating seeds–mature stage | Whole-plant respiration  vs.  whole-plant fresh mass  (Figure 2A, *n* = 306) | Eq. 1 |  | 554.77 | 565.95 | 0.3913  (2.477×10^-2^) |  |  | 0.8133  (1.068×10^-2^) |  |  |
|  |  | **Eq. 2** | **Convex upward** | **433.08** | **451.70** |  | **2.066×10^9^**  (6.177×10^9^) | **0.353**  (1.856×10^-2^) |  | **3.491**  (0.3465) | **0.7626**  (1.049×10^-2^) |
|  |  | Eq. 3 |  | 479.67 | 498.29 |  | 0.3656  (1.319×10^-2^) | −6.881×10^-4^  (2.051×10^-2^) |  | 0.757  (0.1903) | 8.734×10^-2^  (1.957×10^-2^) |
| Seedlings–mature stage | Whole-plant surface area  vs.  whole-plant fresh mass  (Figure 2B, *n* = 154) | Eq. 1 |  | 10.95 | 20.06 | 1.671 (9.300×10^-2^) |  |  | 0.7806 (9.397×10^-3^) |  |  |
|  |  | **Eq. 2** | **Convex upward** | **−72.63** | **−57.44** |  | **393.1 (448.1)** | **1.314 (6.449×10^-2^)** |  | **1.477 (0.1439)** | **0.6536 (2.472×10^-2^)** |
|  |  | Eq. 3 |  | 14.95 | 30.13 |  | 0.4004 (4.469×10^5^) | 1.270 (4.469×10^5^) |  | 0.780648 (2355) | 0.780641 (742.4) |
| Seedlings–mature stage | Whole-plant respiration  vs.  whole-plant surface area  (Figure 2C, *n* = 152) | **Eq. 1** | **Linear** | **145.95** | **155.02** | **0.1566 (1.212×10^-2^)** |  |  | **0.909 (0.01872)** |  |  |
|  |  | Eq. 2 |  | 149.95 | 165.07 |  | 7.916 (1.078×10^7^) | 0.1598 (4390) |  | 0.9087 (8226) | 0.9086 (166.1) |
|  |  | Eq. 3 |  | 149.95 | 165.07 |  | 0.2257 (9.828×10^4^) | −6.903×10^-2^ (9.828×10^4^) |  | 0.90864 (737.0) | 0.90863 (2408) |

Equation 1: ln *Y* = ln *F*+*f* ln *M*. Equation 2: ln *Y* = −ln [1/(*GM^g^*)+ 1/(*HM^h^*)]. Equation 3: ln *Y* = ln (*GM^g^*+ *HM^h^*). Fitting analysis was performed using the nlsLM. The numbers in parentheses indicate the standard error of the mean for each parameter. The model with the lowest AIC value is highlighted in bold. Note that the linear line for whole-plant respiration versus whole-plant surface area shown in Figure 2C represents the results obtained by reduced major axis (RMA) regression in Eq. 1.
