# Supplementary material for: Ontogenetic changes in root and shoot respiration, fresh mass and surface area of Fagus crenata
Source: Ann Bot. 2022 Dec 26;131(2):313–22. doi: 10.1093/aob/mcac143 (PMC9992930; doi:10.1093/aob/mcac143)
Supplement: mcac143_suppl_Supplementary_Table_S4 [file mcac143_suppl_supplementary_table_s4.docx]

Table S4. Results of reduced major axis (RMA) regression (Equation 1: ln *Y* = ln *F*+*f* ln *M*) showing the relationships between whole-plant respiration and whole-plant fresh mass for each provenance using the data of plants within the weight range of 0.001 kg–0.1 kg (Figure S3).

| Provenance | *n* | Range of whole-plant fresh mass (kg) | Slope | 95% CI of Slope | Intercept | 95% CI of Intercept | *R*^2^ |
| --- | --- | --- | --- | --- | --- | --- | --- |
| Iwate | 10 | 0.00106–0.0428 | 0.927 | 0.705–1.08 | 0.632 | 0.254–1.21 | 0.930 |
| Kochi | 14 | 0.00121–0.058 | 0.866 | 0.722–0.953 | 0.652 | 0.295–1.00 | 0.959 |
| Nagano | 19 | 0.00141–0.0157 | 1.00 | 0.662–1.24 | 1.16 | 0.177–4.40 | 0.713 |
| Shizuoka | 8 | 0.00273–0.0767 | 0.843 | 0.750–0.926 | 0.540 | 0.350–0.741 | 0.983 |
| Yamagata | 96 | 0.00103–0.07 | 0.816 | 0.754–0.865 | 0.427 | 0.292–0.571 | 0.873 |
