# Supplementary material for: Ontogenetic changes in root and shoot respiration, fresh mass and surface area of Fagus crenata
Source: Ann Bot. 2022 Dec 26;131(2):313–22. doi: 10.1093/aob/mcac143 (PMC9992930; doi:10.1093/aob/mcac143)
Supplement: mcac143_suppl_Supplementary_Table_S5 [file mcac143_suppl_supplementary_table_s5.docx]

Table S5. Results of fitting analysis and goodness of fit (AIC and BIC statistics) for the scaling relationships among respiration rates, fresh mass, and surface area, for the shoot and root using the dataset of the individuals of seedlings–mature stage (Figure 3A–C).

| Relationship | Equation | Trend | AIC | BIC | *F* | *G* | *H* | *f* | *g* | *h* |
| --- | --- | --- | --- | --- | --- | --- | --- | --- | --- | --- |
| Shoot surface area  vs. shoot fresh mass  (Figure 3A, *n* = 157) | **Eq. 1** | **Linear** | **−44.96** | **−35.79** | **1.432 (7.504×10^-2^)** |  |  | **0.8001 (7.882×10^-3^)** |  |  |
|  | Eq. 2 |  | −42.865 | −27.58 |  | −2.728×10^5^ (4.584×10^6^) | 1.478 (9.229×10^-2^) |  | 2.041 (2.019) | 0.8116 (1.793×10^-2^) |
|  | Eq. 3 |  | −40.96 | −25.68 |  | 1.306 (8.998×10^4^) | 0.1264 (8.998×10^4^) |  | 0.80016 (1891) | 0.80014 (1.953×10^4^) |
| Root surface area  vs. root fresh mass  (Figure 3A, *n* = 164) | Eq. 1 |  | 304.33 | 313.63 | 1.760 (0.250) |  |  | 0.834 (2.103×10^-2^) |  |  |
|  | **Eq. 2** | **Convex upward** | **98.41** | **113.91** |  | **856.0 (511.9)** | **0.5075 (5.683×10^-2^)** |  | **1.591 (6.782×10^-2^)** | **0.4773 (3.428×10^-2^)** |
|  | Eq. 3 |  | 308.33 | 323.83 |  | 0.1878 (5.027×10^5^) | 1.571 (5.027×10^5^) |  | 0.8343749 (1.524×10^5^) | 0.8344745 (1.822×10^4^) |
| Shoot respiration rate  vs. shoot fresh mass  (Figure 3B, *n* = 268) | **Eq. 1** | **Linear** | **354.25** | **365.02** | **0.3778 (1.988×10^-2^)** |  |  | **0.7729 (8.574×10^-3^)** |  |  |
|  | Eq. 2 |  | 356.93 | 374.88 |  | 3651 (5.169×10^4^) | 0.3757 (2.017×10^-2^) |  | 1.677 (1.685) | 0.7607 (2.069×10^2^) |
|  | Eq. 3 |  | 357.02 | 374.98 |  | 0.376 (2.023×10^-2^) | −5.46×10^-5^ (8.201×10^-4^) |  | 0.7617 (2.123×10^-2^) | −8.437×10^-2^ (1.753) |
| Root respiration rate  vs. root fresh mass  (Figure 3B, *n* = 267) | Eq. 1 |  | 284.04 | 294.80 | 0.1841 (9.616×10^-2^) |  |  | 0.7352 (8.487×10^-3^) |  |  |
|  | Eq. 2 |  | 288.04 | 305.98 |  | 0.5146 (5.242×10^5^) | 0.2867 (1.627×10^5^) |  | 0.73520 (355.3) | 0.73519 (1.979×10^3^) |
|  | **Eq. 3** | **Convex downward** | **254.02** | **271.95** |  | **0.0759 (2.861×10^-2^)** | **0.1175 (2.071×10^-2^)** |  | **1.253 (0.1437)** | **0.6723 (2.287×10^-2^)** |
| Shoot respiration rate  vs. shoot surface area  (Figure 3C, *n* = 155) | Eq. 1 |  | 165.11 | 174.23 | 0.2553 (2.449×10^-2^) |  |  | 0.9552 (1.931×10^2^) |  |  |
|  | **Eq. 2** | **Convex upward** | **158.24** | **173.46** |  | **4.114 (9.758)** | **0.2261 (3.808×10^-2^)** |  | **1.379 (0.3384)** | **0.7532 (0.1443)** |
|  | Eq. 3 |  | 169.11 | 184.32 |  | 1.496×10^-2^ (2.329×10^4^) | 0.2404 (2.329×10^4^) |  | 0.9553 (1702) | 0.9552 (105.2) |
| Root respiration rate  vs. root surface area  (Figure 3C, *n* = 162) | Eq. 1 |  | 246.75 | 256.01 | 5.927×10^-2^ (6.218×10^-3^) |  |  | 0.7509 (2.05×10^-2^) |  |  |
|  | Eq. 2 |  | 250.75 | 266.18 |  | 0.2984 (4.337×10^5^) | 7.397×10^-2^ (2.665×10^4^) |  | 0.7509 (1498) | 0.7508 (371.5) |
|  | **Eq. 3** | **Convex downward** | **156.54** | **171.98** |  | **0.2044 (3.607×10^-2^)** | **6.248×10^-3^ (3.096×10^-3^)** |  | **1.319 (0.1094)** | **0.4144 (6.557×10^-2^)** |

Equation 1: ln *Y* = ln *F*+*f* ln *M*. Equation 2: ln *Y* = −ln [1/(*GM^g^*)+ 1/(*HM^h^*)]. Equation 3: ln *Y* = ln (*GM^g^*+ *HM^h^*). Fitting analysis was performed using the nlsLM. The numbers in parentheses indicate the standard error of the mean for each parameter. The model with the lowest AIC value is highlighted in bold. Note that the linear line for shoot respiration rate vs. shoot fresh mass shown in Figure 4A represents the results obtained by reduced major axis (RMA) regression in Eq. 1.
