# Supplementary material for: Ontogenetic changes in root and shoot respiration, fresh mass and surface area of Fagus crenata
Source: Ann Bot. 2022 Dec 26;131(2):313–22. doi: 10.1093/aob/mcac143 (PMC9992930; doi:10.1093/aob/mcac143)
Supplement: mcac143_suppl_Supplementary_Table_S6 [file mcac143_suppl_supplementary_table_s6.docx]

Table S6. Results of fitting analysis and goodness of fit (AIC and BIC statistics) for scaling of the shoot and root respiration, fresh mass, and surface area in relation to the whole-plant fresh mass using the dataset of individuals of seedlings–mature stage (Figure 4A–C).

| Dependent variable | Equation | Trend | AIC | BIC | *F* | *G* | *H* | *f* | *g* | *h* |
| --- | --- | --- | --- | --- | --- | --- | --- | --- | --- | --- |
| Shoot respiration (Figure 4A, *n* = 267) | Eq. 1 |  | 449.91 | 460.67 | 0.2556 (1.534×10^-2^) |  |  | 0.8046 (1.094×10^-2^) |  |  |
|  | Eq. 2 |  | 453.91 | 471.85 |  | 53.42 (8.716×10^7^) | 0.2569 (2015) |  | 0.8048 (5567) | 0.8046 (26.78) |
|  | **Eq. 3** | **Convex downward** | **398.40** | **416.34** |  | **0.2697 (0.01476)** | **5.290×10^-7^ (1.590×10^-6^)** |  | **0.8641 (0.01617)** | **−0.9181 (0.3925)** |
| Root respiration (Figure 4A, *n* = 267) | Eq. 1 |  | 373.17 | 383.93 | 7.801×10^-2^ (4.055×10^-3^) |  |  | 0.6901 (9.479×10^-3^) |  |  |
|  | **Eq. 2** | **Convex upward** | **321.72** | **339.66** |  | **2.703×10^4^ (7.890×10^4^)** | **0.07451 (3.532×10^-3^)** |  | **2.347 (0.3810)** | **0.6384 (1.414×10^-2^)** |
|  | Eq. 3 |  | 330.98 | 348.91 |  | 7.565×10^-2^ (3.662×10^-3^) | −8.730×10^-5^ (1.986×10^-4^) |  | 0.6373 (1.917×10^-2^) | −0.1621 (0.2629) |
| Shoot fresh mass (Figure 4B, *n* = 337) | Eq. 1 |  | 288.67 | 300.13 | 0.588 (2.325×10^-2^) |  |  | 1.031 (6.842×10^-3^) |  |  |
|  | Eq. 2 |  | 292.67 | 311.77 |  | 449.6 (3.423×10^8^) | 0.589 (587.8) |  | 1.03094 (60170) | 1.03090 (78.86) |
|  | **Eq. 3** | **Convex downward** | **50.51** | **69.61** |  | **0.634 (1.784×10^-2^)** | **1.23×10^-4^ (1.061×10^-4^)** |  | **1.126 (1.016×10^-2^)** | **−0.0903 (0.113)** |
| Root fresh mass (Figure 4B, *n* = 337) | Eq. 1 |  | 510.85 | 522.31 | 0.3141 (1.726×10^-2^) |  |  | 0.9478 (9.513×10^-2^) |  |  |
|  | **Eq. 2** | **Convex upward** | **186.94** | **206.04** |  | **2.563×10^4^ (22480)** | **0.2782 (9.546×10^-3^)** |  | **2.499 (0.117)** | **0.8247 (1.062×10^-2^)** |
|  | Eq. 3 |  | 514.85 | 533.95 |  | 8.530×10^-4^ (899.3) | 0.3133 (899.3) |  | 0.947796 (4.730×10^5^) | 0.947795 (1288) |
| Shoot surface area (Figure 4C, *n* = 157) | Eq. 1 |  | 163.54 | 172.71 | 0.6521 (5.82×10^-2^) |  |  | 0.7739 (1.513×10^2^) |  |  |
|  | Eq. 2 |  | 167.54 | 182.82 |  | 352.3 (5.691×10^8^) | 0.6532 (1956) |  | 0.7739 (3.563×10^4^) | 0.7738 (66.14) |
|  | **Eq. 3** | **Convex downward** | **77.88** | **93.16** |  | **0.9561 (0.07685)** | **4.363×10^-4^ (5.732×10^-4^)** |  | **0.946 (3.035×10^-2^)** | **−0.2180 (0.1738)** |
| Root surface area (Figure 4C, *n* = 164) | Eq. 1 |  | 448.02 | 457.32 | 0.8749 (0.1822) |  |  | 0.8238 (3.478×10^2^) |  |  |
|  | **Eq. 2** | **Convex upward** | **257.54** | **273.03** |  | **6.137×10^5^ (7.179×10^5^)** | **0.3128 (4.193×10^-2^)** |  | **2.760 (0.1616)** | **0.4374 (3.744×10^-2^)** |
|  | Eq. 3 |  | 452.02 | 467.52 |  | 0.5897 (1.088×10^6^) | 0.2852 (1.088×10^6^) |  | 0.823777 (1.240×10^4^) | 0.823775 (2.564×10^4^) |

Equation 1: ln *Y* = ln *F*+*f* ln *M*. Equation 2: ln *Y* = −ln [1/(*GM^g^*)+ 1/(*HM^h^*)]. Equation 3: ln *Y* = ln (*GM^g^*+ *HM^h^*). Fitting analysis was performed using the nlsLM. The numbers in parentheses indicate the standard error of the mean for each parameter. The model with the lowest AIC value is highlighted in bold.
