# Supplementary material for: Ontogenetic changes in root and shoot respiration, fresh mass and surface area of Fagus crenata
Source: Ann Bot. 2022 Dec 26;131(2):313–22. doi: 10.1093/aob/mcac143 (PMC9992930; doi:10.1093/aob/mcac143)
Supplement: mcac143_suppl_Supplementary_Table_S7 [file mcac143_suppl_supplementary_table_s7.docx]

Table S7. Results of reduced major axis (RMA) regression (Equation 1: ln *Y* = ln *F*+*f* ln *M*) for scaling of the shoot and root respiration, fresh mass, and surface area in relation to the whole-plant fresh mass for each provenance using the data of plants within the weight range of 0.001 kg–0.1 kg (Figure S4*a*–*f*).

| Dependent variable | Provenance | *n* | Range of whole-plant fresh mass (kg) | Slope | 95% CI of slope | Intercept | 95% CI of intercept | *R*^2^ |
| --- | --- | --- | --- | --- | --- | --- | --- | --- |
| Respiration rate |  |  |  |  |  |  |  |  |
| Shoot (Figure S4*a*) | Iwate | 10 | 0.00106–0.0428 | 0.970 | 0.753–1.11 | 0.577 | 0.225–1.08 | 0.936 |
|  | Kochi | 14 | 0.00121–0.058 | 0.984 | 0.789–1.11 | 0.618 | 0.207–1.15 | 0.934 |
|  | Nagano | 19 | 0.00141–0.0157 | 1.240 | 0.715–1.57 | 2.180 | 0.136–14.8 | 0.661 |
|  | Shizuoka | 8 | 0.00273–0.0767 | 0.912 | 0.73–1.09 | 0.413 | 0.166–0.76 | 0.950 |
|  | Yamagata | 96 | 0.00103–0.07 | 0.936 | 0.849–1.01 | 0.434 | 0.251–0.661 | 0.767 |
|  |  |  |  |  |  |  |  |  |
| Root (Figure S4*d*) | Iwate | 10 | 0.00106–0.0428 | 0.869 | 0.502–1.2 | 0.111 | 0.0234–0.482 | 0.776 |
|  | Kochi | 14 | 0.00121–0.058 | 0.749 | 0.655–0.814 | 0.167 | 0.1–0.223 | 0.965 |
|  | Nagano | 19 | 0.00141–0.0157 | 0.708 | 0.557–0.853 | 0.104 | 0.0468–0.23 | 0.779 |
|  | Shizuoka | 8 | 0.00273–0.0767 | 0.801 | 0.567–0.916 | 0.185 | 0.0832–0.309 | 0.946 |
|  | Yamagata | 96 | 0.00103–0.07 | 0.750 | 0.665–0.822 | 0.134 | 0.0794–0.207 | 0.859 |
| Fresh mass |  |  |  |  |  |  |  |  |
| Shoot (Figure S4*b*) | Iwate | 10 | 0.00106–0.0428 | 1.010 | 0.961–1.09 | 0.631 | 0.47–1 | 0.991 |
|  | Kochi | 14 | 0.00121–0.058 | 1.020 | 0.947–1.06 | 0.403 | 0.264–0.5 | 0.990 |
|  | Nagano | 19 | 0.00141–0.0157 | 1.170 | 1.09–1.27 | 1.030 | 0.689–1.78 | 0.980 |
|  | Shizuoka | 8 | 0.00273–0.0767 | 1.070 | 0.862–1.22 | 0.439 | 0.21–0.943 | 0.984 |
|  | Yamagata | 136 | 0.00103–0.07 | 0.943 | 0.884–1.01 | 0.281 | 0.195–0.432 | 0.913 |
|  |  |  |  |  |  |  |  |  |
| Root (Figure S4*e*) | Iwate | 10 | 0.00106–0.0428 | 0.977 | 0.864–1.06 | 0.353 | 0.186–0.575 | 0.982 |
|  | Kochi | 14 | 0.00121–0.058 | 0.995 | 0.972–1.03 | 0.612 | 0.54–0.755 | 0.995 |
|  | Nagano | 19 | 0.00141–0.0157 | 0.891 | 0.811–0.955 | 0.323 | 0.207–0.456 | 0.979 |
|  | Shizuoka | 8 | 0.00273–0.0767 | 0.969 | 0.922–1.07 | 0.581 | 0.458–0.868 | 0.994 |
|  | Yamagata | 136 | 0.00103–0.07 | 1.070 | 1.02–1.12 | 0.897 | 0.64–1.22 | 0.963 |
| Surface area |  |  |  |  |  |  |  |  |
| Shoot (Figure S4*c*) | Iwate | 0 |  |  |  |  |  |  |
|  | Kochi | 9 | 0.00121–0.058 | 0.804 | 0.407–0.834 | 0.481 | 0.0398–0.541 | 0.989 |
|  | Nagano | 0 |  |  |  |  |  |  |
|  | Shizuoka | 7 | 0.00273–0.0767 | 0.875 | 0.743–0.947 | 0.750 | 0.474–1.02 | 0.985 |
|  | Yamagata | 86 | 0.00103–0.07 | 0.757 | 0.671–0.863 | 0.512 | 0.303–0.98 | 0.871 |
|  |  |  |  |  |  |  |  |  |
| Root (Figure S4*f*) | Iwate | 0 |  |  |  |  |  |  |
|  | Kochi | 12 | 0.00121–0.058 | 0.595 | 0.534–0.812 | 0.569 | 0.406–2.19 | 0.920 |
|  | Nagano | 0 |  |  |  |  |  |  |
|  | Shizuoka | 5 | 0.00273–0.0767 | 0.779 | 0.613–0.898 | 1.080 | 0.425–1.57 | 0.985 |
|  | Yamagata | 85 | 0.00103–0.07 | 0.942 | 0.795–1.06 | 2.920 | 1.2–5.93 | 0.845 |
